# Multiple Levels of Synergistic Collaboration in Termite Lignocellulose Digestion

**DOI:** 10.1371/journal.pone.0021709

**Published:** 2011-07-01

**Authors:** Michael E. Scharf, Zachary J. Karl, Amit Sethi, Drion G. Boucias

**Affiliations:** 1 Entomology and Nematology Department, University of Florida, Gainesville, Florida, United States of America; 2 Department of Entomology, Purdue University, West Lafayette, Indiana, United States of America; Universidade Federal do Rio de Janeiro, Brazil

## Abstract

In addition to evolving eusocial lifestyles, two equally fascinating aspects of termite biology are their mutualistic relationships with gut symbionts and their use of lignocellulose as a primary nutrition source. Termites are also considered excellent model systems for studying the production of bioethanol and renewable bioenergy from 2nd generation (non-food) feedstocks. While the idea that gut symbionts are the sole contributors to termite lignocellulose digestion has remained popular and compelling, in recent years host contributions to the digestion process have become increasingly apparent. However, the degree to which host and symbiont, and host enzymes, collaborate in lignocellulose digestion remain poorly understood. Also, how digestive enzymes specifically collaborate (*i.e.*, in additive or synergistic ways) is largely unknown. In the present study we undertook translational-genomic studies to gain unprecedented insights into digestion by the lower termite *Reticulitermes flavipes* and its symbiotic gut flora. We used a combination of native gut tissue preparations and recombinant enzymes derived from the host gut transcriptome to identify synergistic collaborations between host and symbiont, and also among enzymes produced exclusively by the host termite. Our findings provide important new evidence of synergistic collaboration among enzymes in the release of fermentable monosaccharides from wood lignocellulose. These monosaccharides (glucose and pentoses) are highly relevant to 2^nd^-generation bioethanol production. We also show that, although significant digestion capabilities occur in host termite tissues, catalytic tradeoffs exist that apparently favor mutualism with symbiotic lignocellulose-digesting microbes. These findings contribute important new insights towards the development of termite-derived biofuel processing biotechnologies and shed new light on selective forces that likely favored symbiosis and, subsequently, group living in primitive termites and their cockroach ancestors.

## Introduction

Termites (order Isoptera) are well-known for their roles in nutrient cycling in natural ecosystems, as pests of human crops and structures, and as models for renewable energy systems. The order Isoptera is divided into lower and higher termites based mostly on the presence or absence of protozoan gut symbionts [1]-[3]. However, both higher and lower termites harbor diverse collections of prokaryotic symbionts [2]-[7]. Recent metagenomic and metatranscriptomic studies have corroborated long-standing hypotheses [3]-[7] of gut symbionts playing important roles in lignocellulose digestion in higher [8] and lower [9] termites. However, there is also strong transcriptomic evidence to show that termites themselves produce a battery of cellulases, hemicellulases, and lignases that contribute significantly to lignocellulose digestion [9]-[13]. Some genome sequencing efforts have focused on bacterial endosymboints of protozoan symbionts from lower termites, but found no evidence of lignocellulose digestion capabilities [14], [15]. Similarly, proteomic investigations of the lumen contents of higher termite hindguts revealed limited protein-level evidence of prokaryotic symbiont-assisted digestion relative to that suggested by earlier transcriptomic studies [8], [16].

In an effort to better understand collaborative host *and* symbiont digestion [13] in the lower termite *Reticulitermes flavipes*, we previously identified ca. 200 candidate lignocellulase-coding genes through separate host and symbiont transcriptome sequencing efforts [9], [13]. In the present study, we developed novel monosaccharide detection assays and conducted functional and translational studies with termite host gut and symbiont preparations, as well as recombinant enzymes (two cellulases and a candidate lignase [17]-[19]) derived from the *R. flavipes* host gut transcriptome. Our specific goals were to quantify the degrees of biochemical mutualism between (i) host and symbiont, and (ii) among host enzymes during termite lignocellulose digestion. Our findings reveal the first examples of biochemical synergy in glucose liberation from wood lignocellulose among host termites and their symbionts, and among host enzymes expressed in symbiont-free salivary gland tissue. These findings provide novel and timely evidence that contributes to bioenergy technology development. Moreover, these findings further reveal catalytic tradeoffs that support previously untestable hypotheses relating to the evolution of termite mutualism and sociality.

## Results and Discussion

### Overview

**Figure 1:**
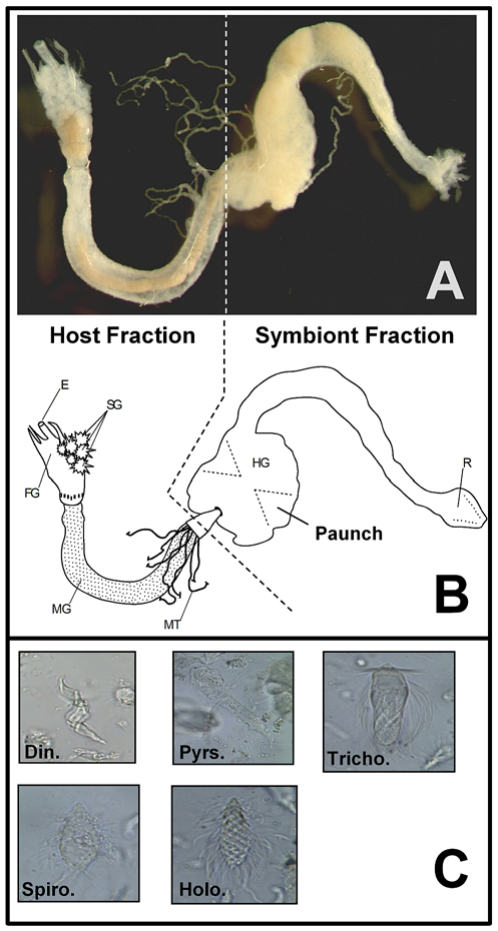
The *R. flavipes* worker-caste digestive tract and common protist gut symbionts. (**A**) Photograph showing host and symbiont fractions used in the current research. (**B**) Drawing of the *R. flavipes* worker digestive tract showing the esophagus (E), salivary glands (SG), foregut (FG), midgut (MG), Malpighian tubules (MT), and hindgut (HG) with the paunch that houses microbial symbionts. Gut regions that served as host and symbiont fractions in the current study are indicated. (**C**) Common protozoan hindgut symbionts of *R. flavipes*: *Dinenympha* (Din.), *Pyrsonympha* (Pyrs.), *Trichonympha* (Tricho.), *Spirotrichonympha* (Spiro.), and *Holomastigotes* (Holo.). Termite gut photo by J.A. Smith.

Softwood lignocellulose is composed mostly of glucose and pentose polymers [20] that are important in termite nutrition [13]. We developed novel approaches that used colorimetric glucose and pentose detection reagents to enable direct and specific quantification of monosaccharide release from pine wood lignocellulose and other cellulosic substrates. In initial validations of these assays, the glucose assay was highly specific to glucose, the pentose assay to xylose and arabinose, and no interference occurred from mono- and disaccharides which can potentially be present in gut homogenates (Fig. S1). To investigate host-symbiont collaboration, we dissected termite guts into host and symbiont fractions and tested them alone or in combination. The host fraction consisted of the salivary gland, foregut and midgut tissues (Fig. 1A, B), while the symbiont fraction consisted of the hindgut and its rich microbial flora (Fig. 1B, C). It is well-established that gut symbionts of lower termites like *R. flavipes* do not reside outside of the hindgut paunch region [2], [3], [7], [13] primarily because of unfavorable oxygen concentrations [13], [19], [21]. In the second half of this study, to determine the contributions of individual host enzymes without competition from co-expressed host and symbiont enzymes, we tested two recombinant cellulases and a laccase that were identified previously and extensively characterized [12], [17]-[19].

### Assays with Native Gut Tissue and Symbiont Fractions

**Figure 2:**
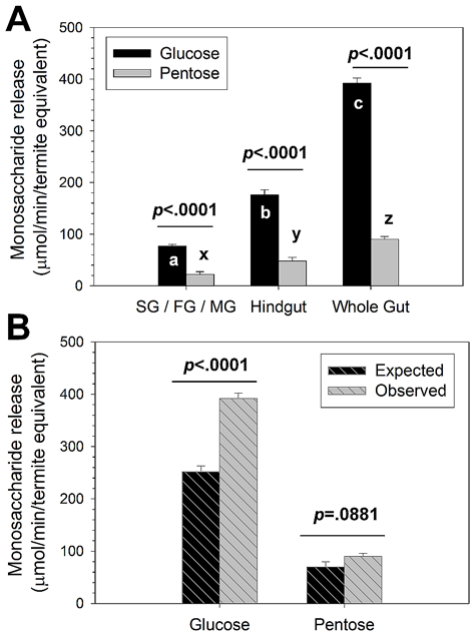
Monosaccharides released from pine lignocellulose by *R. flavipes* host (SG/FG/MG), symbiont (hindgut), and whole gut fractions. (**A**) Glucose and pentose released in 10-hr assays. Bars with different letters (a,b,c or x,y,z) are significantly different by Tukey's HSD test (*p*<0.05). P-values above the bars indicate significant differences in glucose and pentose released within the different gut fractions. (**B**) Comparison of expected and observed glucose and pentose released. Expected values were determined by adding host and symbiont fraction results from (A) above, while observed values are the whole gut results from (A) above. P-values above bars indicate significant differences for expected and observed monosaccharide release. See Tables S1, S2, S3, and S4 for ANOVA summaries. Bars in both panels indicate average ± std. error determined from three colonies with triplicate determinations each.

Wood lignocelluloses, which are considered the main nutritional source of *R. flavipes*, are composed approximately of 40% cellulose, 25% hemicellulose, and 20% lignin. The cellulose fraction is composed of 100% glucose polymers, and the hemicellulose fraction mostly of pentose polymers with <10% glucose content [7], [13], [20]. Thus, glucose can be liberated from both cellulose and hemicellulose, whereas pentoses can only be liberated from hemicellulose. Using pinewood sawdust as a substrate with native gut enzyme preparations, significant glucose liberation was observed in both gut fractions (Fig. 2A; Table S1) indicating significant host and symbiont capabilities in glucose liberation.

While pentose release was also observed in host and symbiont fractions (Fig. 2A; Table S2), it was only released at ∼0.25x levels relative to glucose. The glucose and pentose release levels represent ∼85% and ∼70% release of available monosaccharides, respectively; thus, *not all available glucose and pentose were released and our results do not simply reflect differences between the levels of these two sugars in pine wood*. We therefore conclude that significantly more glucose than pentose release from lignocellulose occurs in the *R. flavipes* gut, suggesting a greater importance for glucose as metabolic currency in both termite and symbiont. These results also correspond strongly with previous transcriptomic findings showing 1.7-fold greater numbers of expressed glucanase/cellulase genes (n = 77) than hemicellulase genes (n = 45) in the *R. flavipes* gut [9].

To investigate if cellulose and hemicellulose digestion occur via additive (*i.e.*, collaborative) or greater-than-additive (*i.e.*, synergistic) processing between host and symbiont, we compared observed vs. expected glucose and pentose released from the different gut fractions (Fig. 2B; Table S3, S4). For this comparison, “observed” results represent monosaccharides released from the whole gut, whereas, “expected” results represent the sum of monosaccharides released from the combined host and symbiont fractions. Observed glucose liberation was significantly greater than expected (about 1.6-fold, *p*<0.0001), while observed and expected pentose released were not different (*p* = 0.0881). Previously, Cleveland [3]-[5] and Hungate [6], based on the earliest experimental evidence of its kind, developed the first hypotheses that host and symbiont may collaborate in termite lignocellulose digestion. While our experimental design cannot account for host-derived enzymes being present in the symbiont (hindgut) fraction, the vastly different oxygen levels *in situ* [21], gene expression profiles [9], [12], [17]-[19], and activity differences (Fig. 2) do not suggest host enzymes contribute significantly to observed activity in the symbiont fraction or *vice versa*. These findings therefore provide the first direct mechanistic evidence of digestive synergy between host and symbiont as proposed by Hungate and Cleveland [3]-[6], but only with respect to glucose liberation (*i.e.*, pentose liberation is additive between host and symbiont fractions, not synergistic).

### Assays with Recombinant Host Enzymes

To further resolve host digestion capabilities as revealed by native tissue assays, and to determine the contributions of individual host enzymes without interference from competing host and symbiont enzymes, we tested three recombinant host enzymes that included a glycohydrolase family (GHF) 1 β-glucosidase (β-glu) [17], a GHF9 endoglucanase (Cell-1) [18], and a phenol-oxidizing laccase involved in lignin degradation (LacA) [19]. All three enzymes have secretory signal peptides and highest expression in symbiont-free salivary gland tissue, and they catalyze cellulase or phenoloxidase activities [17]-[19], including lignin modification in the case of LacA [19]. The pH optima, temperature stability, and cofactor requirements for Cell-1 and β-glu were determined previously [17], [18]. Preliminary investigations on the recombinant laccase enzyme revealed optimal activity at pH 7, as well as sodium azide stimulation and EDTA inhibition (Fig. S2). Sodium azide and the cofactor hydrogen peroxide [19] could not be used in sawdust assays here because they were found to interfere with the glucose detection assay (results not shown).

**Figure 3:**
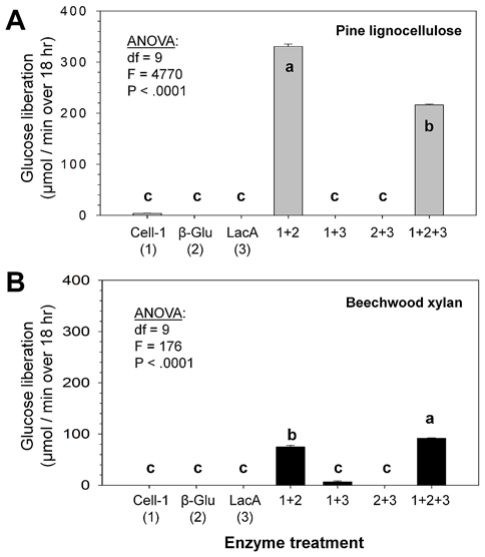
Glucose released from pine lignocellulose and beechwood xylan by three recombinant enzymes that represent genes sampled from the *R. flavipes* host gut transcriptome. (**A**) Pine wood contains a combination of cellulose, hemicellulose and lignin; whereas (**B**) beechwood xylan contains only hemicellulose and lignin. The three recombinant enzymes tested were (1) the Cell-1 endoglucanase, (2) the β-glu beta-glucosidase, and (3) the LacA laccase (see text for details). Each enzyme was tested alone and in two- and three-way combinations. As shown in (A), >300-fold synergy in glucose release occurred when combining Cell-1 and β-glu, but output was reduced in the presence of LacA, likely via end-product inhibition. As shown in (B), Cell-1 and β-glu attained >70-fold synergy when combined, and the addition of LacA resulted in greater glucose output, presumably via lignin-hemicellulose dissociation. Bars within graphs with the same letters are not significantly different by Tukey's HSD test (*p*<0.05). Whole-model ANOVA results indicating significance are shown.

**Figure 4:**
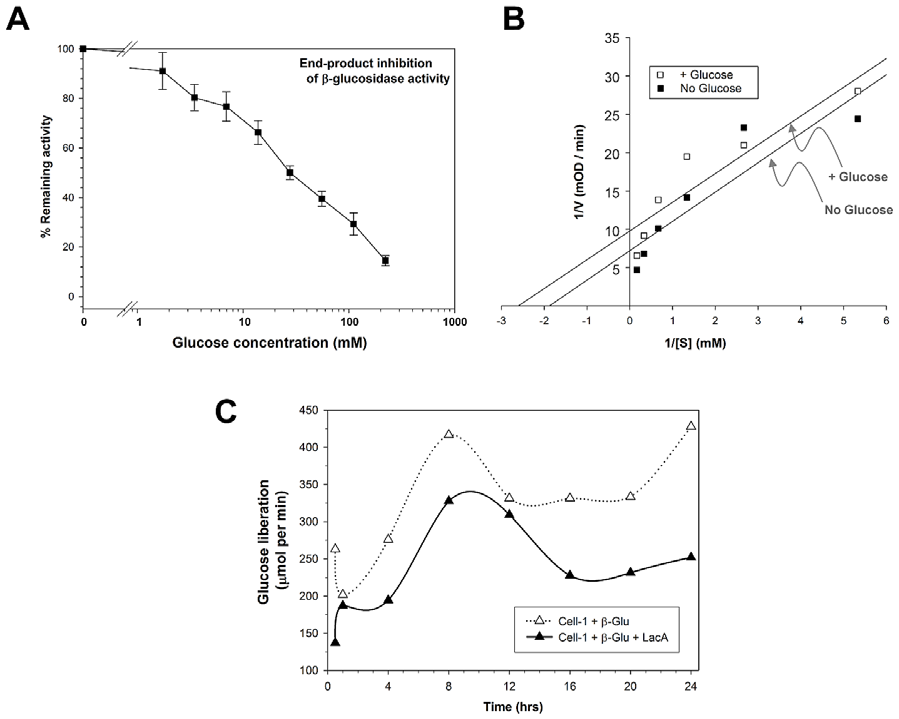
Evidence of end-product inhibition that limits host digestive capabilities. (**A**) Glucose inhibition of beta-glucosidase activity by the recombinant β-glu enzyme. Results show decreasing turnover of the model substrate p-nitrophenyl-β-D-glucopyranoside (pNPG) in the presence of increasing concentrations of free glucose. (**B**) Lineweaver-Burk double-reciprocal plot showing impacts of 10 mM glucose on pNPG activity by the recombinant β-glu enzyme across a range of pNPG concentrations. Results show a shift in both k_m_ and V_max_, suggesting that glucose un-competitively inhibits β-glu via interaction with the enzyme-substrate complex. (**C**) Results of time-course assays comparing the two enzyme combination of Cell-1+β-glu to the three enzyme combination of Cell-1+ β-glu+LacA. Results show continual glucose output through the 24-hr assays, as well as reduced activity for the three-enzyme combination. Reduced activity for the three enzyme combination presumably occurs through end-product inhibition of β-glu as shown in (A) and (B). Combined results from (A), (B) and (C) suggest a host catalytic deficiency that is offset by symbiont cellulose and hemicellulose digestive capabilities.

Quite unexpectedly, when combined, recombinant Cell-1 and β-glu showed >300-fold and >70-fold increases in amounts of glucose released from pine sawdust and beechwood xylan, respectively, relative to each enzyme alone (Fig. 3A, 3B). It was also noteworthy that the three-enzyme combination of Lac-6 + Cell-1 + β-glu released a smaller amount of glucose from pine sawdust than the two-enzyme combination of Cell-1 + β-glu (Fig. 3A), likely through the process of end-product inhibition of β-glu by liberated glucose (Fig. 4A) [22]. In subsequent kinetic analyses, reductions in β-glu K_m_ and V_max_ values in the presence of glucose suggest that glucose un-competitively inhibits β-glu by binding enzyme-substrate complex [23] (Fig. 4B). Additional time-course assays further corroborated the reduced activity for the Cell-1+ β-glu+LacA combination over the two enzyme (Cell-1+ β-glu) combination, as well as provided evidence showing that the two- and three enzyme cocktails remain active for at least 24 hr at 37°C (Fig. 4C).

Converse to pine sawdust assays, the three-enzyme combination of Lac-6 + Cell-1 + β-glu released greater glucose from beechwood xylan than the Cell-1 + β-glu combination (Fig.3B), indicating that Lac-6 significantly enhances glucose release from hemicellulose by host cellulases. Thus, the two host cellulases show a high degree of synergistic collaboration in cellulose and hemicellulose digestion, and additionally host laccase action can enhance glucose release from hemicellulose by host endoglucanases and β-glucosidases, apparently by enabling hemicellulose disassociation from lignin or esterified mono-lignols [13]. When using highly insoluble microcrystalline cellulose as a substrate, results were nearly identical to those obtained with pine sawdust (Fig. S3A). However, the synthetically modified model substrate carboxymethylcellulose, which is amorphous, lignin-free, and frequently used in animal cellulase research, provided very different results suggesting artificially elevated activity (Fig. S3B). Finally, virtually no pentose release was observed from pine sawdust when using the various combinations of recombinant enzymes (Fig. S4), indicating that Cell-1 and β-glu are specific to β-1,4 glycosidic bonds residing between glucose residues.

### Mechanistic and Evolutionary Implications

While unexpected, the findings presented here are highly important. Our results show: (i) greater glucose rather than pentose released from pine lignocellulose (the same food source used to provision termite lab colonies) by native termite gut tissues; (ii) significant synergy between host and symbiont in glucose, but not pentose, released; and (iii) significant glucose release capabilities from various forms of cellulose and lignocellulose by three recombinant host enzymes. As noted above, glucose-pentose release ratios observed here are in strong agreement with ratios of expressed cellulase and hemicellulase genes identified through previous *R. flavipes* transcriptome analysis [9], and there is no evidence to suggest upstream-acting host enzymes contribute significantly to the observed activity in our symbiont preparations, or *vice-versa*.

Most notably, these results show that the host-derived Cell-1 and β-glu enzymes are capable of an unprecedented high degree of synergy in glucose liberation. However, when adding the lignin-phenolic degrading LacA enzyme, glucose liberation by Cell-1 and β-glu was significantly reduced, apparently by end-product inhibition (Fig. 4), or potentially through the process of β-glu-dependent transglucosylation that leads to glucose polymerization and/or conjugation to phenolic compounds [24], [25]. *Therefore, our findings reveal important catalytic costs to lignin dissociation/degradation (e.g., end-product inhibition) that could be a core evolutionary factor favoring termite-symbiont mutualism*. In this respect, hosting a diverse suite of hindgut symbionts that produce synergistic cellulases to overcome end-product inhibition [9], [13] clearly offers an important fitness advantage. Another advantage of hosting cellulose-digesting symbionts is that laccases can act on phenolic precursors of insect cuticle (*e.g.*, tyrosine and dopamine [19]); thus, the over-production of host laccases can potentially disrupt cuticle melanization, a process important in the formation of the intima lining the foregut and hindgut [26]. Lignin degradation also generates free radicals and other metabolites that may be toxic to microbial symbionts [7]. Thus, lignin modifying/degrading capabilities, while enhancing availability of fermentable monosaccharides, could potentially limit the ability of the host termites to maintain its gut physiology and gut microenvironment.

Our data therefore suggest that host gut laccase/lignase/phenoloxidase capabilities should exist in fine balance and dynamic equilibrium with symbiont and other host digestion capabilities. Alternatively, maintenance of host digestive machinery would also be evolutionarily favored not only because it maximizes digestive outputs, but also because symbionts are lost during molting (*i.e.*, symbionts must be replenished by colony mates after each molt, which is facilitated by a social lifestyle). From a socio-evolutionary perspective, our findings provide some of the first mechanistic evidence supporting the hypothesis that, because it favored group living, utilization of a nutritionally poor food source like lignocellulose was a driving force in the social evolution of termites and their pre-social cockroach ancestors [27]–[29].

### Conclusions

Despite early evidence suggesting that termite lignocellulose digestion occurs as a collaboration between host and symbionts [3]–[6], the idea that this process is mediated exclusively by hindgut symbionts has proliferated and remained popular [2], [4], [8]. Our findings provide important new evidence showing that digestion capabilities of the host termites are significant, and also that host and symbiont synergistically contribute to release of the fermentable monosaccharide glucose from lignocellulose in the termite gut. These findings provide unique and novel glimpses into termite digestion and host-symbiont mutualism, and they are highly relevant from basic and applied perspectives. Whether host termites acquired their intrinsic capabilities of lignocellulose digestion after adapting to a eusocial lifestyle and/or successful establishment of symbiosis is a tantalizing question that will remain unresolved until further molecular evolutionary analysis of their symbiosis.

From the basic perspective of termite social evolution, by revealing catalytic and nutritional tradeoffs, these findings provide new support of previously untestable hypotheses on termite and cockroach social living and host-symbiont mutualism [27]–[29]. From an applied perspective, by revealing synergistic enzyme combinations and synergistic outputs, our findings contribute important new information to assist in the development of novel biocatalyst technologies and strategies for producing bioethanol from 2nd generation (non-food) feedstocks [30], [31]. In particular, it will now be possible to test a wide range of recombinant symbiont cellulases and hemicellulases [9], [13] in combination with the host enzymes reported here as synergistic biocatalyst cocktails for use in industrial biomass-to-bioethanol operations.

## Materials and Methods

### Termites

*R. flavipes* colonies were collected in Gainesville, FL, USA and maintained in sealed plastic boxes (30×24×10 cm) in complete darkness (0:24 L:D), at 22°C and 70%RH. Colonies were maintained without soil for 1–3 months before use and provisioned with moist brown paper towels and pine wood shims (Nelson Wood Shims; Cohasset, MN). The identity of colonies as *R. flavipes* was verified by a combination of soldier morphology and 16S-mt-rDNA gene sequences. Only worker termites were used because of their significant lignocellulose digestion capability relative to other castes.

### Dissections and Native Gut Preparations

Gut dissections were made from worker termites sampled from three *R. flavipes* laboratory colonies. Two gut fractions from 25 individual workers per colony were collected: (i) foregut + salivary gland + midgut ( =  host fraction), and (ii) hindgut ( =  symbiont fraction). The gut fractions were pooled separately in phosphate buffered saline, pH 7.0. Tissues were manually grounded using 1.5 mL Pellet Pestles (Kimble-Kontes; Vineland, NJ), centrifuged for 15 min at 14,000 *xg* and 4°C, and the supernatant collected. To assure consistency, protein content was determined using a commercial Bradford assay (Bio-Rad; Hercules, CA). Gut preparations were used at 5 termite equivalents per digestion assay per replicate. The host and symbiont fractions were combined in assays at 5 termite equivalents each to provide whole-gut conditions.

### Recombinant Enzymes and Digestion Assays

Three recombinant enzymes were prepared by Chesapeake-PERL Inc. (Savage, MD, USA) and tested in highly purified form. These included the *β-glu* beta-glucosidase [17] (Genbank Accession No. HM152540), the *Cell-1* endoglucanase [18] (Genbank Accession No. AY572862), and the *LacA* laccase [19] (Genbank Accession No. GQ421909). Each enzyme was expressed, purified, and extensively characterized in preceding studies as cited above. Cell-1 and β-glu were tested at 8 µg per assay. LacA was tested at 1 µg per assay based on preliminary determinations. Assays contained 2% w/v substrate in 750 µL sodium acetate buffer (0.1M, pH 7) containing 0.01 M calcium chloride. Substrates used were pine sawdust (Nelson Wood Shims; Cohasset, MN), beechwood xylan, microcrystalline cellulose, and carboxymethyl cellulose (Sigma-Aldrich). The pH of the various substrate reaction mixtures, determined using a pH meter and pH indicator paper, ranged from 6.4–6.6. Assays with either native gut preparations or recombinant enzymes (see quantities above) were incubated in vented 1.5-mL tubes at 37°C and 220 rpm for either 10 hr (native tissue assays) or 18–22 hr (recombinant enzyme assays). Assays were stopped with 0.2 M EDTA at a ratio of 10 µL per 500 µL assay buffer volume, to provide a final concentration of 4 mM. The addition of EDTA in this manner was found to greatly stabilize color formation after glucose and pentose detection (see following sections). Reaction tubes were centrifuged 5 min at 16,000 xg, and the resulting supernatant used for glucose and pentose detection as described below.

### Glucose Detection

A commercial glucose-mutarotase (glucose C2) detection reagent was used for glucose detection (Wako Chemical; Richmond, VA). Supernatants from digestion assays were obtained as described above and quantified in 96-well microplates. Each assay technical replicate was divided into three 50-µL reaction aliquots, which were each used for glucose detection. Reagents for the glucose assay were added at 200-µL per well, followed by orbital shaking for 5 mins. Absorbance was read as an endpoint at 505 nm relative to a glucose standard curve. Each assay plate was run in real-time with its own glucose standard curve. All assays included buffer blanks that contained all reaction components except protein. Standard curves were prepared as seven serial dilutions in assay buffer with 4 mM EDTA from 5 mM downward to 0.078125 mM. The 8^th^ well that contained only buffer served as the blank control.

Several mono- and disaccharides were assayed in standard curve format to validate the specificity of the glucose assay. The mono- and disaccharides tested included glucose, mannose, galactose, xylose, arabinose, rhamnose, glucuronic acid, galacturonic acid, cellobiose, sucrose, and trehalose (Sigma-Aldrich; St. Louis, MO). Serial dilutions (8–12) from 5 mM downward were tested in the validation assays (Fig. S1). Serial dilutions were made in assay buffer with 4 mM EDTA. The assay was highly specific to glucose and showed only minimal mannose interference outside the linear glucose detection range (Fig. S1A,C).

### Pentose Detection

This assay employed the methods of Roe and Price [32] and Deschaletes and Yu [33]. The initial step was preparation of bromoaniline detection reagent. All procedures were performed in a fume hood with appropriate safety precautions. First, in a 15-mL screw-cap plastic vial, 15-mL glacial acetic acid (Fisher Scientific; Suwanee, GA) was added to 0.6-g thiourea (Sigma-Aldrich). This saturated solution was gently inverted several times and centrifuged 5 mins at 1,000xg. The supernatant was transferred to a 50 mL screw-cap plastic tube and the thiourea pellet saved for future re-use. Next, 0.3-g of 4-bromoaniline (Sigma-Aldrich) was added to the supernatant and gently inverted 3–4x. This created enough reagent for 75 replicate microplate assay wells. The reagent had a yellowish color and remained active for over 1 month when kept wrapped in foil at room temperature.

For pentose detection, supernatants from digestion assays were obtained as described above and transferred to microplate wells. Three reaction aliquots were quantified per assay technical replicate using 50-µL per well. Bromoaniline detection reagent was added at 200-µL per well. The plate was then covered, placed within a Pyrex baking dish, and heated at 70°C in a drying oven for 15 min. Next, the plate (still within the Pyrex dish) was covered with aluminum foil and left to cool to room temperature in a fume hood for 70 min. Absorbance was read as an endpoint at 520 nm relative to a xylose standard curve. All assay runs included buffer blanks that contained all reaction components except protein. Each assay plate was run in real-time with its own xylose standard curve. Standard curves were prepared as seven serial dilutions in assay buffer + 4 mM EDTA from 5 mM downward to 0.078125 mM. An 8^th^ well that contained buffer alone served as a blank.

The same mono- and disaccharides noted above for glucose detection were assayed in standard curve format to validate pentose assay specificity. Twelve serial dilutions from 5 mM downward were tested in the validation assays. Serial dilutions were made in assay buffer with 4 mM EDTA. This assay was highly specific to the pentose sugars xylose and arabinose, with minimal interference from galactose at 2.5 mM and above (Fig. S1B,D).

### Glucose (End-Product) Inhibition of Beta-glucosidase Activity

Beta-glucosidase activity assays followed the methods of Scharf et al. [17], using the model substrate p-nitrophenyl-β-D-glucopyranoside (pNPG; Sigma-Aldrich). It was not possible to use natural substrates and glucose detection reagent in these glucose-inhibition assays because of interference from glucose. Assays took place in 0.1 M sodium acetate buffer (pH 7), using 1.58-µg (2-µL) of recombinant β-glu [17] in a total reaction volume of 250-µL. To examine end-product inhibition by glucose, recombinant protein (2-µL) was pre-incubated with 50-µL buffer plus glucose solution for 5 min at room temp. Eight serial dilutions of glucose were tested (250, 125, 62.5, 31.25, 15.63, 7.81, 3.91, and 1.95 mM), plus an uninhibited control. Reactions were initiated by adding 200-µL assay buffer containing 2.4 mM pNPG to the 50-µL mixtures of protein + glucose. Assays were read kinetically at 420 nm every 20 sec for 5 min to yield mean velocity data in mOD/min. Activity was determined based on the p-nitrophenol extinction coefficient of 0.6605 mM^−1^cm^−1^. Glucose inhibition of beta glucosidase activity was further tested using a single glucose concentration of 10 mM across a serial dilution range of substrate concentrations (6, 3, 1.5, 0.75, 0.372, 0.1875 mM), with analysis by the Lineweaver-Burk double reciprocal method [23]. Reported results were averaged from three independent replicates, each conducted in triplicate. Finally, time-course assays as shown in Fig. 4C were conducted up to 24 hr using double (Cell-1+ β-glu) and triple (Cell-1+ β-glu+LacA) recombinant enzyme mixtures, with glucose detection as described above.

### Replication Strategies and Statistical Analyses

Native gut tissue assays were run using three independent colonies, each with three gut preparations and triplicate determinations for each. Recombinant enzyme assays were performed using three independent replicates, each with triplicate determinations. Statistical analyses consisted of one-way analysis of variance (ANOVA), followed by mean separation using Tukey's HSD test (*p*<0.05) only when ANOVA model statements were first determined significant.

## Supporting Information

Figure S1 **Standard curves.** Standard curves used to validate the specificity of **(A, C)** glucose and **(B, D)** pentose detection reagents. Eight monosaccharides (glucose, mannose, galactose, xylose, arabinose, rhamnose, glucuronic acid, galacturonic acid) and three disaccharides (cellobiose, sucrose, trehalose) were tested. Glucose and xylose were included for reference in C and D.
(DOCX)

Figure S2 **Recombinant laccase characterizations.** pH dependence (A), and sodium azide enhancement and EDTA inhibition (B) for the recombinant Lac6/A laccase against the model substrate 2,6-dimethoxyphenol (DMP; Coy *et al*., 2010. *Insect Biochemistry and Molecular Biology* 40: 723-732). Sawdust reaction buffer [0.1 M sodium acetate containing 0.01 M calcium chloride and 50 mM hydrogen peroxide] was used in both assays. (A) shows strongly enhanced activity in pH 7 buffer conditions with 50 mM hydrogen peroxide. (B) Shows activity levels at pH 7 for untreated Lac6/A, sodium azide-enhanced Lac6, and EDTA-inhibited Lac6/A.
(DOCX)

Figure S3 **Glucose release from microcrystalline cellulose and carboxymethyl cellulose by three recombinant enzymes that represent genes sampled from the** ***R. flavipes*** **host gut transcriptome.** (**A**) Microcrystalline cellulose is highly insoluble and is a dominant natural form of lignocellulose; whereas (**B**) carboxymethyl cellulose is a chemically modified, highly soluble, synthetic version of cellulose. The three recombinant enzymes tested were the Cell-1 endoglucanase (1), the β-glu beta-glucosidase (2), and the LacA laccase (3) (see text and Fig. 3 for details). Each enzyme was tested alone and in two- and three-way combinations. As shown in (A), >10-fold synergy in glucose release occurred when combining Cell-1 and β-glu, but output was reduced in the presence of LacA. As shown in (B), much higher activity for individual enzymes was observed with no evidence suggesting synergy (contrast against A and Fig. 1). Bars within graphs with the same letters are not significantly different by Tukey's HSD test (*p*<0.05). Whole-model ANOVA results indicating significance are shown.
(DOCX)

Figure S4 **Pentose release from pine lignocellulose by three recombinant enzymes that represent genes sampled from the** ***R. flavipes*** **host gut transcriptome.** Glucose release (black bars) is shown for reference. See *Materials and Methods* and preceding figures for methodological details. Results show expected degrees of glucose release, but virtually no pentose release, indicating that Cell-1 and β-glu are highly specific to β-1,4 glucose linkages.
(DOCX)

Table S1 **Glucose ANOVA, native gut fractions.**
(DOCX)

Table S2 **Pentose ANOVA, native gut fractions.**
(DOCX)

Table S3 **ANOVA for observed vs. expected glucose release by native gut fractions.**
(DOCX)

Table S4 **ANOVA for observed vs. expected pentose release by native gut fractions.**
(DOCX)
