# Supplementary material for: Multiple Levels of Synergistic Collaboration in Termite Lignocellulose Digestion
Source: PLoS One. 2011 Jul 1;6(7):e21709. doi: 10.1371/journal.pone.0021709 (PMC3128603; doi:10.1371/journal.pone.0021709)
Supplement: Figure S1 — Standard curves. Standard curves used to validate the specificity of (A, C) glucose and (B, D) pentose detection reagents. Eight monosaccharides (glucose, mannose, galactose, xylose, arabinose, rhamnose, glucuronic acid, galacturonic acid) and three disaccharides (cellobiose, sucrose, trehalose) were tested. Glucose and xylose were included for reference in C and D. (DOCX) [file pone.0021709.s001.docx]

**Fig. S1.** Standard curves used to validate the specificity of **(A, C)** glucose and **(B, D)** pentose detection reagents. Eight monosaccharides (glucose, mannose, galactose, xylose, arabinose, rhamnose, glucuronic acid, galacturonic acid) and three disaccharides (cellobiose, sucrose, trehalose) were tested. Glucose and xylose were included for reference in C and D.
