# Supplementary material for: Multiple Levels of Synergistic Collaboration in Termite Lignocellulose Digestion
Source: PLoS One. 2011 Jul 1;6(7):e21709. doi: 10.1371/journal.pone.0021709 (PMC3128603; doi:10.1371/journal.pone.0021709)
Supplement: Figure S2 — Recombinant laccase characterizations. pH dependence (A), and sodium azide enhancement and EDTA inhibition (B) for the recombinant Lac6/A laccase against the model substrate 2,6-dimethoxyphenol (DMP; Coy et al., 2010. Insect Biochemistry and Molecular Biology 40: 723-732). Sawdust reaction buffer [0.1 M sodium acetate containing 0.01 M calcium chloride and 50 mM hydrogen peroxide] was used in both assays. (A) shows strongly enhanced activity in pH 7 buffer conditions with 50 mM hydrogen peroxide. (B) Shows activity levels at pH 7 for untreated Lac6/A, sodium azide-enhanced Lac6, and EDTA-inhibited Lac6/A. (DOCX) [file pone.0021709.s002.docx]

**Fig. S2.** Recombinant laccase characterizations. pH dependence (A), and sodium azide enhancement and EDTA inhibition (B) for the recombinant Lac6/A laccase against the model substrate 2,6-dimethoxyphenol (DMP; Coy *et al*., 2010. *Insect Biochemistry and Molecular Biology* 40: 723-732). Sawdust reaction buffer [0.1 M sodium acetate containing 0.01 M calcium chloride and 50 mM hydrogen peroxide] was used in both assays. (A) shows strongly enhanced activity in pH 7 buffer conditions with 50 mM hydrogen peroxide. (B) Shows activity levels at pH 7 for untreated Lac6/A, sodium azide-enhanced Lac6, and EDTA-inhibited Lac6/A.
