# Supplementary material for: Multiple Levels of Synergistic Collaboration in Termite Lignocellulose Digestion
Source: PLoS One. 2011 Jul 1;6(7):e21709. doi: 10.1371/journal.pone.0021709 (PMC3128603; doi:10.1371/journal.pone.0021709)
Supplement: Figure S3 — Glucose release from microcrystalline cellulose and carboxymethyl cellulose by three recombinant enzymes that represent genes sampled from the R. flavipes host gut transcriptome. (A) Microcrystalline cellulose is highly insoluble and is a dominant natural form of lignocellulose; whereas (B) carboxymethyl cellulose is a chemically modified, highly soluble, synthetic version of cellulose. The three recombinant enzymes tested were the Cell-1 endoglucanase (1), the β-glu beta-glucosidase (2), and the LacA laccase (3) (see text and Fig. 3 for details). Each enzyme was tested alone and in two- and three-way combinations. As shown in (A), >10-fold synergy in glucose release occurred when combining Cell-1 and β-glu, but output was reduced in the presence of LacA. As shown in (B), much higher activity for individual enzymes was observed with no evidence suggesting synergy (contrast against A and Fig. 1). Bars within graphs with the same letters are not significantly different by Tukey's HSD test (p<0.05). Whole-model ANOVA results indicating significance are shown. (DOCX) [file pone.0021709.s003.docx]

**Fig. S3.** Glucose release from microcrystalline cellulose and carboxymethyl cellulose by three recombinant enzymes that represent genes sampled from the *R. flavipes* host gut transcriptome. **(A)** Microcrystalline cellulose is highly insoluble and is a dominant natural form of lignocellulose; whereas **(B)** carboxymethyl cellulose is a chemically modified, highly soluble, synthetic version of cellulose. The three recombinant enzymes tested were the Cell-1 endoglucanase (1), the β-glu beta-glucosidase (2), and the LacA laccase (3) (see text and Fig. 3 for details). Each enzyme was tested alone and in two- and three-way combinations. As shown in (A), >10-fold synergy in glucose release occurred when combining Cell-1 and β-glu, but output was reduced in the presence of LacA. As shown in (B), much higher activity for individual enzymes was observed with no evidence suggesting synergy (contrast against A and Fig. 1). Bars within graphs with the same letters are not significantly different by Tukey’s HSD test (*p*<0.05). Whole-model ANOVA results indicating significance are shown.
