# Supplementary material for: Multiple Levels of Synergistic Collaboration in Termite Lignocellulose Digestion
Source: PLoS One. 2011 Jul 1;6(7):e21709. doi: 10.1371/journal.pone.0021709 (PMC3128603; doi:10.1371/journal.pone.0021709)
Supplement: Figure S4 — Pentose release from pine lignocellulose by three recombinant enzymes that represent genes sampled from the R. flavipes host gut transcriptome. Glucose release (black bars) is shown for reference. See Materials and Methods and preceding figures for methodological details. Results show expected degrees of glucose release, but virtually no pentose release, indicating that Cell-1 and β-glu are highly specific to β-1,4 glucose linkages. (DOCX) [file pone.0021709.s004.docx]

**Fig. S4**. Pentose release from pine lignocellulose by three recombinant enzymes that represent genes sampled from the *R. flavipes* host gut transcriptome. Glucose release (black bars) is shown for reference. See *Materials and Methods* and preceding figures for methodological details. Results show expected degrees of glucose release, but virtually no pentose release, indicating that Cell-1 and β-glu are highly specific to β-1,4 glucose linkages.
