# Supplementary material for: Multiple Levels of Synergistic Collaboration in Termite Lignocellulose Digestion
Source: PLoS One. 2011 Jul 1;6(7):e21709. doi: 10.1371/journal.pone.0021709 (PMC3128603; doi:10.1371/journal.pone.0021709)
Supplement: Table S3 — ANOVA for observed vs. expected glucose release by native gut fractions. (DOCX) [file pone.0021709.s007.docx]

**Table S3**. ANOVA for observed vs. expected glucose release by native gut fractions.
